# Replacing an ‘In-Person’ Global Health Annual Conference With a Virtual Format: A Case Study from the Consortium of Universities for Global Health

**DOI:** 10.5334/aogh.3695

**Published:** 2022-03-31

**Authors:** Melissa W. Li, Keith Martin, Joseph C. Kolars

**Affiliations:** 1 University of Michigan Medical School, Ann Arbor, Michigan, US; 2 Consortium of Universities for Global Health, Washington, DC, US

## Abstract

**Background:**

Beginning in 2020, the COVID pandemic disrupted many planned annual meetings that relied on travel to a destination for sharing scholarship, networking, and planning future collaborations. As with many organizations, the Consortium of Universities for Global Health (CUGH) began exploring the utilization of a virtual platform on which to conduct the annual conference.

**Objective:**

We sought to understand the value of conducting an annual conference virtually and to evaluate the added benefit of utilizing a learning management system.

**Methods:**

Routinely collected registration data was used for the CUGH 2021 annual conference, which was completely virtual, and compared to in-person registration data from prior years. In addition, tracking and engagement data from a learning management system was reviewed to understand participation.

**Findings:**

The virtual conference attracted the greatest number of registrations, from the largest number of countries since the organization began in 2008. Analyzing the engagement of participants with specific sessions through the on-line learning management system allowed a deeper understanding of the popularity and value of topics.

**Conclusion:**

A virtual format is an efficient and effective venue for scholarly conferences. The additional information gained from an on-line learning management system can provide valuable information for future conference planning.

## Background

Professional societies rely on annual conferences for scholarly exchange and networking. These gatherings often serve as an important mechanism to engage members, build professional relationships, share knowledge, and generate revenue for the organization. The Consortium of Universities for Global Health [1] (CUGH) began holding annual conferences in 2008 to bring together academic institutions relevant to the interdisciplinary issues related to Global Health. With a membership of 182 academic institutions and a network of over 32,000 global health professionals, CUGH considers the annual conference as one of its major activities.

Attendance has been increasing at the three-day annual meeting with a growing number of preconference, free, open access satellite sessions and affiliated events. However, the COVID-19 pandemic that began in 2019 brought with it world-wide concerns about enhanced transmission of the virus at the meeting or during the travel process. This resulted in the complete cancellation of the annual meeting scheduled for March of 2020. Hopes were placed on restarting the annual conference for March of 2021 in Houston, Texas as originally scheduled three years earlier. However, ongoing concerns among public health authorities warning against unnecessary travel and large group meetings lead the CUGH leadership to replace the planned in-person event with a completely virtual meeting. Concerns among conference planners included the following:

- Would members still attend a purely virtual meeting? How might this impact the learners who are a major component of attendees? Those from outside the U.S. and Canada?
- Would a virtual meeting facilitate attendee engagement in a manner that allowed revenue generation for the organization?
- Would we have other unintended consequences or unanticipated benefits from switching to a completely virtual conference?

In this paper, we review available data relative to these questions that reflect our experience with this conference, *CUGH 2021 Addressing Critical Gaps in Global Health and Development*, which was successfully conducted virtually in March of 2021.

## Methods

CUGH 2021 [2] consisted of two main components that were completely virtual: a collection of 30 satellite symposia held March 1 through March 11, 2021, and the main conference program held March 12 (Friday) through 14 (Sunday), 2021. Sessions were organized as follows with oral, ‘live’, zoom presentations along with pre-recorded ePosters that were available for viewing.

- Global Leader Interviews
- Plenary Sessions
- Concurrent Sessions
- Special Events and General Sessions

Based on content, each session or ePoster was assigned to one of seven thematic tracks (see Appendix 1).

Data that is routinely gathered was reviewed and analyzed from three different sources:

1. Registration information
  Registration data for the annual conference was acquired on-line by the CUGH Secretariat using the software platform Aventri [3]. The data was exported into an Excel spreadsheet. From 22 questions posted to registrants, the acquired data included demographic information such as attendee name, contact information, institution, job title, learner (e.g., student, trainee). This mechanism was also used to collect a registration fee.
2. Post-conference survey
  The routine practice has been to ask every attendee to fill out an on-line, post-conference survey.
3. On-line participation by conference attendees
  The CUGH Secretariat secured on-line management services from The Conference Exchange® (Confex) [4]. This provided a learning management platform for attendees that facilitated registration and engagement with conference sessions, many of which were concurrent, and ePosters. This platform linked attendees to real-time sessions held on Zoom [5] which were also recorded and available for later asynchronous viewing. Because attendees logged in for all activities [6], engagement and participation during the sessions was routinely collected within the learning management platform (>12,000 log-ins) and provided, deidentified as to the individual, to the CUGH Secretariat for analysis.

Data were analyzed by using Microsoft Excel. Summary statistics of conference sessions were derived using the data.table package in the open source R statistical computing environment [7].

## Results

As noted in ***Table 1***, registrations for CUGH 2021 exceeded those of the prior three annual conferences. Historically, the number of registrants who do not attend has been low (< 10%) and for CUGH 2021, 456 (24%) of those who registered did not appear to sign-in to the conference viewing web. The number of learners increased for this conference as well as those attending from outside of the U.S. and Canada.

**Table 1:** Registrations in the Past Four Conferences.


YEAR*	TOTAL REGISTRANTS	LEARNER** % (N)	AMERICAN & CANADIAN REGISTRANTS	NON-AMERICAN, NON-CANADIAN REGISTRANTS % (N)	LMIC REGISTRANTS

2021	1905	33% (637)	1391	27% (514)	297

2019	1659	37% (618)	1398	16% (261)	288

2018	1721	35% (595)	1453	16% (268)	253

2017	1550	29% (450)	1327	14% (223)	152


* CUGH2020 was completely cancelled because of the COVID 19 pandemic. ** Student, trainee (e.g., post-doc, resident, fellow, etc.).

CUGH uses a sliding scale for its conference registration fees. Historically, the highest fee of $600 is charged to non-members from high-income countries and the lowest fee of $115 charged to delegates and students members from low-income countries. All students are charged a reduced rate. After discussions with a number of like-minded organizations that were switching their conferences to a virtual format, a decision was made to reduce registration fees by 25%. Of note, this was the largest reduction in fees that any of the organizations spoken to were willing to entertain.

Historically, responses to the post-conference surveys have been low (12–15%) and therefore of limited utility. Similarly, in 2021 only 15% of the attendees responded to multiple requests electronically for feedback which did not contain any meaningful information for purposes of this report. Of the 1905 individuals registered for the conference, over half (64%) were from the U.S. and Canada, representing 184 institutions, the majority of which are CUGH member institutions. Top participating institutions include Charles Drew University, Tulane University, Stanford University, University of Michigan, University of Illinois, Chicago, and University of California- San Francisco. Compared to previous in-person meetings, the number of registrants from outside the U.S. and Canada was 514 (27%) which is the highest recorded for a CUGH annual meeting. Attendees from LMICs were particularly noteworthy with the largest numbers of attendees from Rwanda, Uganda, South Africa, Haiti, and Nigeria (see Appendix 2).

The 637 learners (i.e., high schoolers, undergraduates, graduates, medical school students, residents, postdoctoral fellows, scholars) at this conference is the highest recorded. They represent 33% of total registrants which is comparable to in-person attendance over the past several years (see ***Table 1***). The majority of learners are from the U.S. (77%) with learners from low- and middle-income countries (LMICs) accounting for only 14% (n = 89).

One of the unintended benefits of the learning management system is the ability to capture attendance at sessions as well as how long participants remain engaged (i.e. ‘signed-in’ or connected to the session). As shown in ***Table 2***, Concurrent Sessions and Plenary Sessions, unsurprisingly, were the most popular although this varied as to the specific session. These two types also offered the greatest number of sessions. Of note, concurrent sessions by definition had competition from other activities relative to the other sessions which explains lower attendance.

When a participant attended a session, they remain connected, on average, for just under half the time (i.e., 46%) The Global Leader Interviews attracted attendees to stay for the longest time (i.e. 71% which translates to just over 40 minutes for a one-hour session). Attendance per day of the week could also be determined and we found that the interview on Sunday, the last day of the conference, had the highest attendance which is different from what some have observed with regard to in-person conferences when the last day has early departures as people return home. (see Appendix 3).

**Table 2:** Attendance and engagement by session type.


TYPE	TOTAL SESSIONS	TOTAL ATTENDANCE	AVERAGE ATTENDANCE PER SESSION	% OF SESSION COMPLETED BY THOSE WHO ATTENDED

Concurrent Sessions	48	3035	63	46%

Global Leader Interviews	5	672	134	71%

Plenary Sessions	10	2465	247	44%

Satellite Symposia	31	4869	157	N/A

Special Events and General Sessions	22	1006	46	34%

**Total**	**116**	**12047**	**104**	46%*


* *Excluding satellite symposia*.

Anaylsis of participation and engagement allowed us to further analyze each of the session types. For example, the 48 concurrent sessions were distributed across seven thematic tracks (see Appendix 3) where *Track 7: Translation and Implementation Science, High Impact Development Initiatives, Bridging Research to Policy, Reforming Academia* attracted the greatest number (i.e. 79 attendees/session) compared to what appeared to be least popular, *Track 5: Strengthening Health Systems, Public Health, Primary and Surgical Care (37 attendees/session)*. Yet track 5 retained the engagement of their attendees (average 53% of session) more than any of the other tracks (see Appendix 4).

Of the ten Plenary Sessions across seven tracks, *Track 2: Covid-19, Emerging Infectious Diseases, & Other Communicable Diseases* attracted the most participants at 413 attendees/session relative to the least attractive *Track 3: Politics, Law, Corruption, Human Rights, Governance, Diplomacy, Strengthening Public Institutions* at 146 attendees/session. On the other hand, Track 2 engaged attendees for less than 40% of the sessions while Track 3 kept attendees for over 70% of the session (see Appendix 5).

The Satellite Symposia took place before the core conference. A total of thirty-one sessions were presented between March 1 and March 11. The average attendance was 157 participants per session. The kickoff session, titled Equitably harnessing the power of health data: Time for Action and Collaboration attracted over 500 people to watch. Other popular sessions were the 9th Annual Symposium on Global Cancer Research (two sessions). Due to the platform setup, no attendance duration data were captured for Satellite Symposia. The full list of Satellite Symposia sessions and attendance are shown in Appendix 6.

Six hundred ninety electronic posters (ePosters) were visited by over 7,000 unique visitors. Each poster was viewed on average by 10 ‘visitors’. Visitor number per poster concentrated in the range between 6–20: 344 posters received 6–10 visitors and 244 posters were viewed by 11–20 visitors. Posters across the tracks received quite even attention with the exception of Track 9, which was for student ePoster competition and increased visits from judges (see Appendix 7).

Another unanticipated benefit of these virtual sessions is to retain recordings of all the zoom sessions. The recordings were posted shortly after the sessions and available to registrants during or after the conference. The views are tracked by the ConFex system. During the conference, video recordings of sessions were accessed for 298 viewings. Within ten days after the conference, 80 sessions out of the total 116 sessions recorded were viewed (total viewings almost 1000). In the subsequent 20 days, 90 recordings had an additional 90 viewings. Total viewings within three months of the conference have numbered 2,580.

## Discussion

Registrations for the CUGH 2021 Conference set a historical record with an increase in those from outside the U.S. and Canada and LMICs as well as learners. Increased registrations have also been noted at other conferences that rely heavily on virtual formats [8,9], suggesting that the absence of an ‘in person’ component may not detract from interest in participation. We speculate that the inconvenience of traveling and associated costs, particularly for those outside of the U.S., was an advantage [10,11]. However, we were surprised to see a ‘no show’ rate of 24%, despite the payment of registration fees, which is higher than observed for previous in-person meetings. The COVID pandemic placed new time demands and pressures on individuals which may have prevented attendance as intended. Other possibilities include technical difficulties signing on to the web-based system although this was not reported to the secretariat. It may be that the absence of opportunities to interact with colleagues and be energized by being physically present (i.e., stopping other activities to immerse oneself in the conference) did in fact detract from decisions by some to participate.

The on-line nature of the learning platform allowed for the collection of data on participation that was not previously available. For past in-person events, actual participation required utilization of RSVPs for a session, informal ‘head-counts’ in a room, or surveys after a session that was typically incomplete. The learning platform utilized for CUGH 2021 allowed a better appreciation of how many people were connecting, and for how long, to each session. This allowed a better appreciation of the relative popularity of types of sessions and the content covered. We were surprised to realize that, on average, participants remain connected to a particular session for less than 50% of the time. Although people leaving part-way through an in-person session, or joining late, has always been the case, to our knowledge this is the first opportunity to measure how long a participant is connected to a session. Whether participants are leaving to join other sessions or connect to other conference activities (e.g., viewing ePosters) is unknown. We also don’t know to what degree participants may have been multi-tasking (e.g., tending to email or other activities) while listening to a session.

Poster sessions are a particularly valuable opportunity for learners, as well as other conference attendees, to display their scholarship so as to obtain input and potential interest in collaboration. Historically, it has been difficult for conference organizers to understand at a quantitative level what is taking place with regard to engagement with the posters. However, from learning platforms such as the one utilized for CUGH 2021, the number of ‘visits’ could be tracked with data that might illuminate which posters or topics attracted more attention than others.

Video-recordings of in-person sessions have long been utilized to extend access to conferences. However, little has gone into evaluating the benefit of these curated resources. With the learning management platform utilized for this conference, all sessions were recorded, and we were able to note 2,580 viewings within the first three months of the conference.

Associations such as the Consortium of Universities for Global Health consider collaboration and engagement with international colleagues, particularly those in LMICs to be of the highest priority. We would like to use the data coming from learning platforms to better understand the engagement of those from LMICs so as to maximize programming that may be of particular benefit.

The reduction in fees charged to registrants was offset from the reduced expenses that arise from utilizing on-site conference facilities. Although some penalties for cancellation of facility obligations were incurred by CUGH, this virtual conference generated a net revenue that exceeded past conferences. Worth emphasizing is the commitment CUGH maintained to subsidize the registration of all learners as well as those from LMICs who made up a sizeable component of the cohort.

This paper has a number of descriptive components to illustrate the kind of data that can be gathered by virtual learning platforms. Limitations are present in addition to questions worthy of pursuing through future scholarship. Much of the data presented in this paper was only available via the virtual platform and lacks comparators from past conferences that would help us to better understand the impact of switching to a completely virtual format. In addition, we lack sufficient information on attendee satisfaction from participating in a virtual conference where the networking and spontaneous conversations that occur with in-person meetings were absent. While theoretically available within the learning platform, we did not explore learning behaviors at the level of the individual. For example, there are likely sub-groups of individuals who were connecting to major major portions of the conference as well as those who only connected for a session or two. For in-person conferences, individuals usually have blocked-off time to travel and attend major portions of a conference whereas those attending virtually may be squeezing in participation between other non-conference related activities. While it may appear that more people are attending the conference, in fact the breadth and depth of that which they are connecting to may be more limited. For the data set from this 2021 conference, individuals could connect from different portals or pathways when signing-on which made it difficult to track what any one individual was doing. Of note, future tracking would need to be done with great caution and respect to the privacy of individuals who increasingly have their on-line behaviors monitored. Finally, the concept of ‘engagement’ with the conference also has limitations because participants may be away from the computer during the session although ‘connected’ or distracted from concomitant multi-tasking. The number of questions raised by an individual or ‘chat comments’ posed via the learning platform will likely be better reflections of engagement.

## Conclusion

The pivot to a completely virtual annual meeting for CUGH in 2021 was deemed successful with an apparent strong record of participation captured by a virtual learning platform with metrics previously unavailable from in-person conferences. Most of the data observations raise questions that will be worthy of future scholarship. To answer many of these questions, improvements in the learning management system will be required along with informed consent by participants. This conference had the greatest geographic diversity amongst attendees which we speculate is due to the lowered barrier to access from the reduced need to travel and perhaps lower registration fees. We still suspect the impact of not having in-person interactions is likely a negative consequence though difficult to measure. On-line learning platforms will provide new insights regarding participant engagement and learning behaviors than what was previously available from in-person conferences. This also allows for continuous quality improvement among conference planning committees in terms of what people are choosing to connect to and possibly why. As noted by management guru Peter Drucker, you can’t improve on that which you cannot measure [12].

## Additional File

The additional file for this article can be found as follows:

10.5334/aogh.3695.s1 Appendices. Appendixs 1 to 7.
