# Supplementary material for: Replacing an ‘In-Person’ Global Health Annual Conference With a Virtual Format: A Case Study from the Consortium of Universities for Global Health
Source: Ann Glob Health. 2022 Mar 31;88(1):23. doi: 10.5334/aogh.3695 (PMC8973777; doi:10.5334/aogh.3695)
Supplement: Appendices. — Appendixs 1 to 7. [file agh-88-1-3695-s1.pdf]

Appendix 1. Conference Tracks

- Track 1: Addressing the Social Determinants of Health
- Track 2: Covid-19, Emerging Infectious Diseases, & Other Communicable Diseases
- Track 3: Politics, Law, Corruption, Human Rights, Governance, Diplomacy, Strengthening Public Institutions
- Track 4: Planetary Health, One Health, Environmental Health, Climate Change and Pollution
- Track 5: Strengthening Health Systems, Public Health, Primary and Surgical Care
- Track 6: Non-Communicable Diseases
- Track 7: Translation and Implementation Science, High Impact Development Initiatives, Bridging Research to Policy, Reforming Academia
- Track 8: Global Health Education Pathway
- Track 9: Student ePoster Competition Candidates

Appendix 2. Countries with ≥ 10 registrants from outside the U.S.*

Canada	81
Rwanda	40
Uganda	40
South Africa	35
Haiti	28
Nigeria	27
United Kingdom	26
India	23
Kenya	19

Ghana	16
Peru	14
Australia	12
Philippines	12
Brazil	11
Mexico	10

* field left blank on registration for 13

Appendix 3 Global Interview Series

Session	Day	Total Attendees
1359 - Corruption, Money Laundering and Global Health with with Tom Burgis, Investigations Correspondent, Financial Times, UK	Sunday	186
1349 - Biodiversity, the Extinction Crisis and Human Health: An interview with Elizabeth Mrema, Executive Secretary of the United Nations Convention on Biological Diversity	Saturday	157
1361 - Addressing Geopolitical Challenges with Richard Haas, President, Council on Foreign Relations	Saturday	150
1334 - Resetting our Relationship with Nature: Improving Human and Environmental Health with Inger Andersen, Under-Secretary-General of the United Nations and Executive Director, United Nations Environment Programme	Friday	150
1360 - Addressing Critical Gaps in Global Health with Muhammad Ali Pate, Global Director, Health, Nutrition and Population Global Practice, The World Bank	Friday	120

Appendix 4 Concurrent Sessions

Track	Total # of Sessions	Total # of Attendees	Average # of Attendees/Session	Average Attendance Duration (%)
Track 1: Addressing the Social Determinants of Health	7	485	69	46%
Track 2: Covid-19, Emerging Infectious Diseases, & Other Communicable Diseases	10	543	54	45%
Track 3: Politics, Law, Corruption, Human Rights, Governance, Diplomacy, Strengthening Public Institutions	5	367	73	47%
Track 4: Planetary Health, One Health, Environmental Health, Climate Change and Pollution	4	200	50	39%
Track 5: Strengthening Health Systems, Public Health, Primary and Surgical Care	4	146	37	53%
Track 6: Non-Communicable Diseases	5	262	52	48%
Track 7: Translation and Implementation Science, High Impact Development Initiatives, Bridging Research to Policy, Reforming Academia	13	1032	79	46%
all tracks combined	***48***	***3035***	***63***	***46%***

Appendix 5. Plenary Sessions

Track	Total # of Sessions	Total # of Attendees	Average # of Attendees/Session	Average Attendance Duration (%)
Track 1: Addressing the Social Determinants of Health<br>-PL02: Addressing the Social Determinants of Health During the Pandemic in Latin America and the Caribbean<br>-PL14: African Leaders: Addressing Critical Gaps in Global Health and Development	2	455	228	46%
Track 2: Covid-19, Emerging Infectious Diseases, & Other Communicable Diseases<br>-PL01: Welcome, Michele Barry, Chair, CUGH and Keith Martin, Executive Director, CUGH and Conference Executive Planning Committee; Keynote Interviews: Anthony Fauci and Hugo Lopez-Gatell<br>-PL10: Women Leaders in the COVID-19 Pandemic Response	2	825	413	37%
Track 3: Politics, Law, Corruption, Human Rights, Governance, Diplomacy, Strengthening Public Institutions	1	146	146	74%
Track 4: Planetary Health, One Health, Environmental Health, Climate Change and Pollution	1	259	259	35%
Track 5: Strengthening Health Systems, Public Health, Primary and Surgical Care	1	173	173	42%
Track 6: Non-Communicable Diseases	1	262	262	46%
Track 7: Translation and Implementation Science, High Impact Development Initiatives, Bridging Research to Policy, Reforming Academia<br>-PL07: Gairdner Global Health Awardees Address<br>-PL12: Newborn Survival: Achieving SDG 3.2 in Less Than 10 Years	2	345	173	51%
all tracks combined	***10***	***2465***	***247***	***44%***

Appendix 6 – Sattelite Symposia

Date	Day	Total Attendees	Satellite Symposia Title	Session Attendees
3/1/21	Monday	537	SA18 Equitably harnessing the power of health data: Time for Action and Collaboration	537
3/2/21	Tuesday	687	SA9 Implementation Science for Global Health: Building Research Capacity<br>SA14 Facing COVID-19: How Big Food & Big Tobacco undermined public health<br>SA29 The Importance of Noncommunicable Diseases in Pandemic Times	236<br>215<br>236
3/3/21	Wednesday	378	SA8 Resiliency in the Global Health Workforce During the COVID-19 Pandemic<br>SA16 Regional and global perspectives on climate change and health: focusing on solutions<br>SA15 Advocating for Children & Families Globally in the COVID Era and Beyond	168<br>118<br>92
3/4/21	Thursday	397	SA25 Tele Medicine: a sustainable and cost-effective health care solution for rural areas<br>SA1 Approaches to Addressing Gaps in Health Research in Conflict Settings I<br>SA27 Part 1: Asset Mapping To Bridge Gaps And Empower Communities In The Surgical Ecosystem: Holding Space To Keep Our Oath<br>SA13 Advancing Global Health through Cross-Cutting Approaches in Stigma Reduction Research	213<br>84<br>0<br>100
3/5/21	Friday	330	SA5 AFREhealth and CUGH: Implementing the goals of a collaborative partnership<br>SA27.5 Part 2: Asset Mapping To Bridge Gaps And Empower Communities In The Surgical Ecosystem: Holding Space To Keep Our Oath	119<br>211
3/6/21	Saturday	154	SA10 Education Committee Satellite Conference<br>SA2 Mobile health (mHealth) for Enhancing Access to Affordable and Efficient Care at Remote and Underserved Locations in India and Translating Lessons Learnt to a Global Platform	99<br>55
3/8/21	Monday	430	SA21 Strategies to Address Global Health Inequities<br>SA24 Building Capacity for Global Cancer Prevention, Diagnosis and Treatment<br>SA33 Gun Violence in Mexico and Central America: The Challenges and the Paths to Solutions	187<br>102<br>141
3/9/21	Tuesday	400	SA20 Creating exceptional women leaders: the missing links of mentorship and networking<br>SA26 Nursing and Midwifery Leadership in the Global Health Context: Lessons from Africa<br>SA32 Closing the gap between knowledge and practice: implementation science priorities for health adaptation for climate change<br>SA7 Global Health in Hawai'i and the U.S-Affiliated Pacific Islands	167<br>82<br>0<br>151
3/10/21	Wednesday	710	SA6.0 Part 1: 9th Annual Symposium on Global Cancer Research<br>SA3 Women Leaders in Global Health: Exploring Non-Academic Careers and Strengthening Networks<br>SA11 Advocating for Global Health While at Home: How Trainees can be Effective Advocates	372<br>233<br>105
3/11/21	Thursday	846	SA6.5 Part 2: 9th Annual Symposium on Global Cancer Research<br>SA22 Utilizing Technology to Expand Capacity for Health Education<br>SA23 Increasing Health Equity by confronting racism, bias and discrimination in Health Workforce Education and Global Health<br>SA19 Impact of Climate Change on Health in the Caribbean, and Health Opportunities from Climate Action across the North American Region.<br>SA31 Global Ideas for Local Challenges: Networking to Advance Health Equity<br>SA4 9th Annual Houston Global Health Collaborative Conference	308<br>242<br>149<br>56<br>91<br>0

Appendix 7 ePosters

Track	Total # of Posters	Total # of Unique Visitors	Average # of Visitors/Poster
Electronic Poster Track 1: Addressing the Social Determinants of Health	94	1064	11
Electronic Poster Track 2: Covid-19, Emerging Infectious Diseases, & Other Communicable Diseases	87	934	11
Electronic Poster Track 3: Politics, Law, Corruption, Human Rights, Governance, Diplomacy, Strengthening Public Institutions	19	180	9
Electronic Poster Track 4: Planetary Health, One Health, Environmental Health, Climate Change and Pollution	25	297	12
Electronic Poster Track 5: Strengthening Health Systems, Public Health, Primary and Surgical Care	151	1247	8
Electronic Poster Track 6: Non-Communicable Diseases, Emerging Infectious Diseases & Other Communicable Diseases	100	810	8
Electronic Poster Track 7: Translation and Implementation Science, High Impact Development Initiatives, Bridging Research to Policy, Reforming Academia	53	488	9
Electronic Poster Track 8: Global Health Education Pathway	137	1558	11
Electronic Poster Track 9: Student ePoster Competition Candidates	24	495	21
**all tracks**	***690***	***7073***	***10***
